# Gene3D: Extensive prediction of globular domains in proteins

**DOI:** 10.1093/nar/gkx1069

**Published:** 2017-11-03

**Authors:** Tony E Lewis, Ian Sillitoe, Natalie Dawson, Su Datt Lam, Tristan Clarke, David Lee, Christine Orengo, Jonathan Lees

**Affiliations:** Institute of Structural and Molecular Biology, Division of Biosciences, University College London, Gower Street, London WC1E 6BT, UK; School of Biosciences and Biotechnology, Faculty of Science and Technology, University Kebangsaan Malaysia, 43600 Bangi, Selangor, Malaysia; Bristol Life Sciences Building, University of Bristol, Bristol Life Sciences Building, Bristol, BS8 1TQ, UK; Oxford Brookes University, Faculty of Health and Life Sciences, Oxford, Oxfordshire, UK

## Abstract

Gene3D (http://gene3d.biochem.ucl.ac.uk) is a database of globular domain annotations for millions of available protein sequences. Gene3D has previously featured in the Database issue of *NAR* and here we report a significant update to the Gene3D database. The current release, Gene3D v16, has significantly expanded its domain coverage over the previous version and now contains over 95 million domain assignments. We also report a new method for dealing with complex domain architectures that exist in Gene3D, arising from discontinuous domains. Amongst other updates, we have added visualization tools for exploring domain annotations in the context of other sequence features and in gene families. We also provide web-pages to visualize other domain families that co-occur with a given query domain family.

## INTRODUCTION

Sequence and structural databases continue to expand rapidly, with the current version of UniProtKB (v2017_08) containing nearly 90 million sequences (1). Globular domains are one of the fundamental units of evolution and can be organised into (sometimes very large) homologous families.

The CATH database (2) identifies the distinct globular domains in the PDB and then classifies each of these, using sequence and structural features, into one of a few thousand families. Gene3D uses profile-Hidden Markov Models built from the CATH domain sequences to predict structural domains for proteins. The last step in the Gene3D assignment pipeline is to resolve any overlapping domain assignments to provide a final domain-architecture with no overlaps between domains.

There are several important improvements in the current release of Gene3D v16 compared to the v14 published previously (3). We have moved our domain assignments to the latest release of CATH (v4.2) and improved the HMM building process, providing greater coverage of sequence space. We have added to the website comparative genomics tools for displaying domains in gene trees and changes in domain family partners in proteins across different genomes.

### Updates to the Gene3D database

#### Domain annotations

The previous release of Gene3D (v14) (3) made use of CATH v4.1 and updated the HMM building process to using five rounds of jackhmmer, from the HMMER package (4) to build its HMM profiles. Although this can improve sensitivity, successive iteration searches can also lead to divergence from the original family. To help minimize this, we have restricted the number of rounds of jackhmmer to between one and two. We benchmark both of these models against the known CATH domain assignments as our gold standard, to decide on which one to use. We use the first round by default, but switch to the second round HMM if the number of True Positives increases (with no increase in False Positives) or the number of False Positives decreases (with no decrease in True Positives). In future releases, we will explore ways in which further rounds of jackhmmer can be deployed, exploring strategies discussed elsewhere (5).

The CATH resource provides different domain sequence clusterings (e.g. S35, S60, S95) using complete-linkage clustering where the number represents the minimum sequence identity that all members of a cluster must share (hence S95 clusters are finer grained than S30 clusters). We have updated our v16 pipeline to build HMM’s for all S95 models, compared to using only S35 HMMs in v14, resulting in an increase in the number of HMMs (from 21 000 to over 59 000).

Despite the more conservative approach in building the HMMs, Gene3D v16 has improved sequence coverage of domain assignments compared to v14. Mapping between releases, using a shared set of Ensembl genomes sequences and scaling to the equivalent search space (based on the -Z hmmsearch parameter set to 10 000 000 and an independent e-value cut-off of 0.001, which we now apply as our default in Gene3D v16 (Gene3D v14 ran hmmscan with no *Z*-score parameter set), we find that the number of sequences covered by at least one domain annotation has grown by 5% in Gene3D v16 relative to Gene3D v14. Most important to the increase in sequence coverage is the CATH update from v4.1 to v4.2, expanding the number of S35 domain sequences from 21 155 to over 32 000.

We have updated the underlying datasets in Gene3D such as the Ensembl genomes. In addition, we have added proteomes using the set of UniProt Reference Proteomes. This includes a number of viral proteomes, not covered by Ensembl, such as medically important viruses. The total number of protein sequences annotated with a domain in the Gene3D database has greatly expanded to over 52 million sequences.

Through genomic re-arrangements, domains can be inserted into other domains with important consequences for function, such as altering catalytic properties (6). CATH domain choppings systematically record domain insertion events, sometimes identifying quite complex cases where discontinuous domains have themselves been inserted into other domains. When inspecting Gene3D assignments, we noticed that the standard HMMs built from individual domains found some domain insertion patterns difficult to predict. To help cope with this, we have updated our domain assignment strategy by building additional HMMs that cover the entire sequence region of the discontinuous domain (i.e. combining the sequence of the domain that is inserted into and the domain that is inserted). This makes sense from the point of view that the domains, once undergoing an insertion event, can become evolutionarily stable units. Benchmarks using CATH showed there to be improvements in discontinuous domain prediction through these models, especially for complex discontinuous domain insertion patterns, with the discontinuous HMMs providing a 5% improvement in the percentage of correctly identified discontinuous domains.

Apart from the improvements to the HMM-building pipeline, we have also updated the domain resolution algorithm, to a new method we have developed called **c**ath-**r**esolve-**h**its (CRH). As mentioned in the introduction, predicting domains for a query protein involves scanning the protein sequence against profile-HMM libraries and then resolving the candidate matches to obtain a final set of non-overlapping domain assignments (i.e. the final domain architecture). The scans typically assign a score (e.g. bit-score or e-value) to the candidate matches and this can be used to prioritise strong matches in cases of domain overlaps. CRH uses dynamic programming instead of a more computationally expensive graph-based method. It is faster, more flexible and at least as accurate as the previous DomainFinder3 method (7). CRH is available via the cath-tools resource pages (http://cath-tools.readthedocs.io/). The new Gene3D annotation pipeline is easy to run, memory efficient and can be downloaded along with the HMMs from the FTP site (ftp://orengoftp.biochem.ucl.ac.uk/gene3d/CURRENT_RELEASE/gene3d_hmmsearch.tar.gz).

#### Additional data and updates

As in the previous release we added CATH-FunFam annotations produced by the FunFHMMer pipeline (8). In this release we also provide Pfam-FunFam annotations. These annotations were shown to be valuable for functional prediction (9). We have expanded our structural annotations to include eight extra organisms, from the two included in the previous release (including mouse and fission yeast genomes). Other resources such as Gene Ontology annotations, protein interaction networks and DrugBank data have all been updated. Finally, we have added tissue, cell line and cancer-specific gene expression and proteomics datasets from the Human Protein Atlas to the Gene3D database, which can provide additional functional information for a protein.

### Website updates

#### Protein domain visualisation

**Figure 1.**
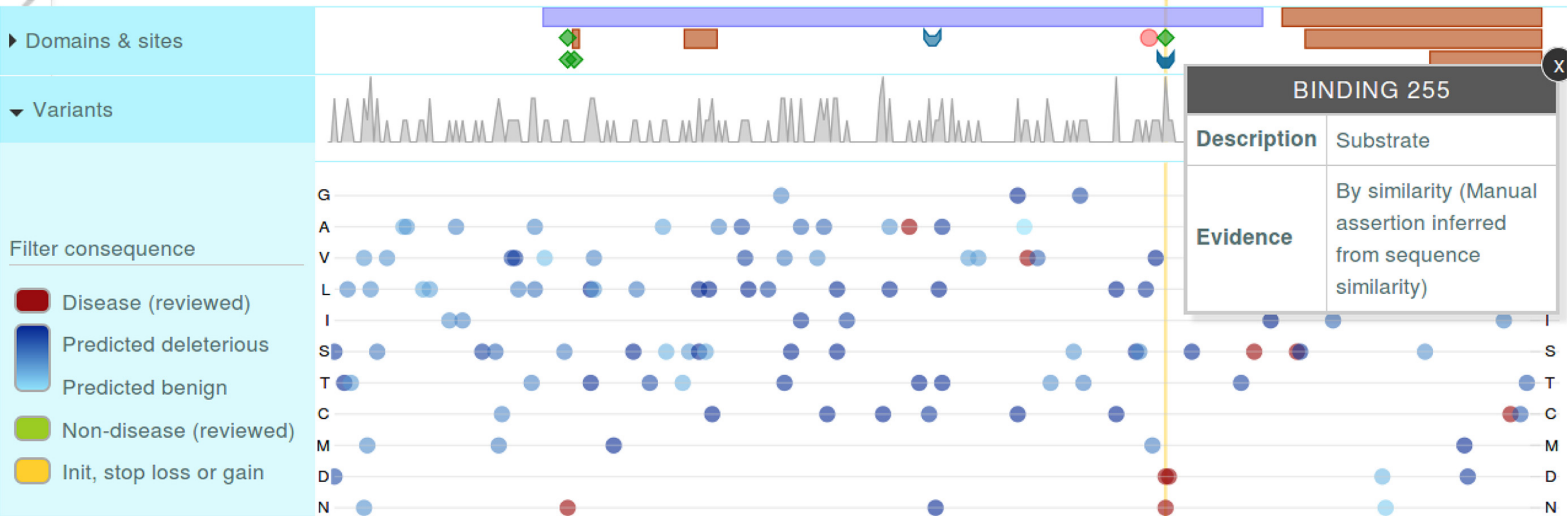
Gene3D domains are now displayed along with other sequence annotations using the ProtVista tool.

In the following sections we discuss some of the new visualization features that make use of the database updates described above. As an example, in the protein page view, we have added the ProtVista tool (10) which allows our in house domain assignments to be visualized along with a range of complementary sequence features. Combining domain annotations with other sequence features in this way can help with interpreting both sets of data. For example, for the human protein TREX1_HUMAN, we find no domains returned by the default ProtVista domain annotation resource, but adding in the Gene3D domains allows us to see that this protein has a domain with superfamily 3.30.420.10 (FunFam:Three-prime repair exonuclease 1), that overlaps an active site residue, which in turn is near to a mutation associated with Aicardi-Goutieres Syndrome (annotated to reduce activity by 75%) (Figure 1).

The protein sequence viewer now also optionally displays CATH-PDB domain architectures (i.e. direct CATH choppings of the PDB mapped onto the protein sequence by SIFTS (11)), allowing us to check how the Gene3D domain assignments compare to the known annotations contained in CATH. For example, we can see how our discontinuous domain predictions compare to those contained in CATH, to assess discontinuous domain architecture predictions (Supplemental Figure S1A and B). To avoid too much information being displayed at once, the CATH-PDB assignments are hidden by default and the user is presented with the option to display them when they are available for a protein.

#### Protein domain visualisation in gene trees

Genes can be grouped into families, and gene trees can be built from these, identifying speciation and duplication events at different points in the tree. Various resources exist that build gene trees and some now provide this data in PhyloXML format (http://www.phyloxml.org) to download or access via web-services. For a given protein coding gene from Ensembl, we can now visualise its globular domains relative to those of other genes from the same Ensembl Compara (12) gene family in the Gene3D website. Viewing domain architectures in this manner is useful for a number of tasks, including identifying missed domain assignments, potential gene annotation artifacts and also genuine domain architecture changes. To do this we have made use of the PhyD3 JavaScript library (13), which allows us to display domain architectures for a gene family along with duplication events and their associated confidence values. PhyD3 allows us to easily display the domains of each gene at the leaf nodes of the tree, pop-ups are available for each tree node.

**Figure 2.**
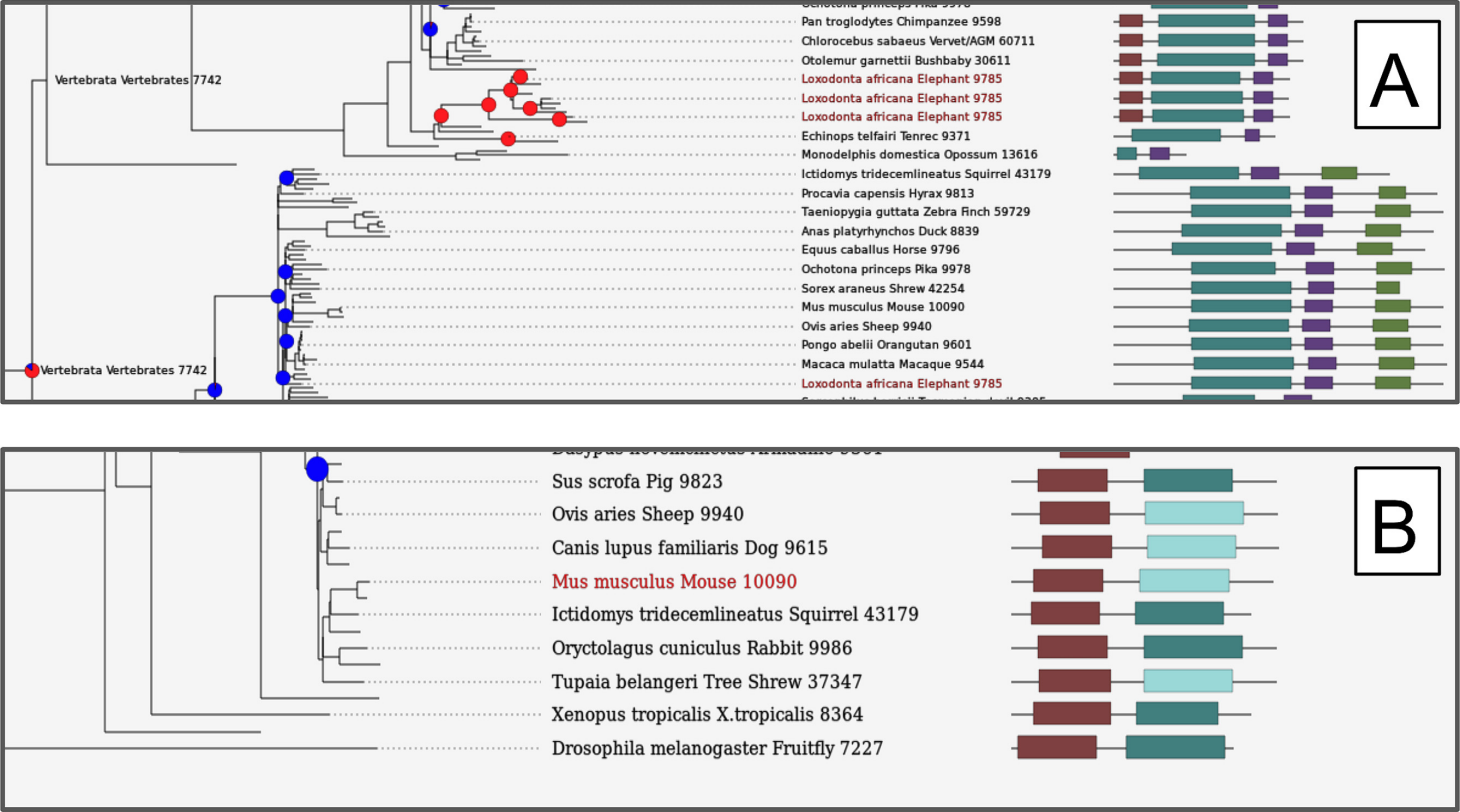
The Gene3D website displaying protein domain architectures in a gene tree, using the PhyD3 JavaScript library. (**A**) A query with an elephant P53 gene, zoomed in on the area of recurrent P53 duplication, shows multiple paralogs with similar architectures. P63 genes with altered domain architectures can be seen in the lower half of the figure as part of the same gene tree. (**B**) A Gene3D search with mouse Trpt1, shows it has a non-significant hit to domain superfamily 3.20.170.30 (light blue rectangles) whilst other members of the gene family have a significant hit to this same domain family (darker blue–green rectangles).

Figure 2 shows two examples, firstly of the domain architectures for the gene family containing P53 genes (Figure 2A) and secondly for a gene associated with regulator of kinase activity (Figure 2B). In the first example, we search with Ensembl gene:ENSLAFG00000027474, an elephant P53 gene. We can see a number of features, such as large-scale gene duplication of P53 in elephants producing a number of paralogs (Figure 2A) (which has relevance to cancer suppression in these large, long-lived mammals (14)). In the tree, we also see a high confidence vertebrate duplication event, leading to different groups of genes within the gene tree with different domain architectures (i.e. P53 and P63 sub-groups of the tree).

For the second example, we show the mouse Trpt1 protein (Ensembl gene: ENSMUSG00000047656), a regulator of kinase activity, along with other genes from its Ensembl Compara gene family. By default, Gene3D filters domain annotations with a HMMER independent e-value < 0.001. However HMMER also provides domain predictions measured by a conditional e-value and we allow these domains to be optionally displayed in the tree by adjusting the domain confidence filter bar, and display these low confidence predictions in a lighter color (light blue in Figure 2B). In our example for Trpt1, changing the confidence filter in the website, reveals that mouse Trpt1 has a low confidence domain from amino acids 122–208 with CATH superfamily 3.20.170.30 (from the ADP-ribosylation fold). Notably, many other gene family relatives of mouse Trpt1 also have this same superfamily predicted, in a similar C-terminal location, but with significant independent e-value, providing some support for the low confidence domain prediction of superfamily 3.20.170.30 for mouse Trpt1. The gene tree views can be displayed using alternative domain sources, such as FunFams and Pfam.v30.

#### Domain architecture pages

**Figure 3.**
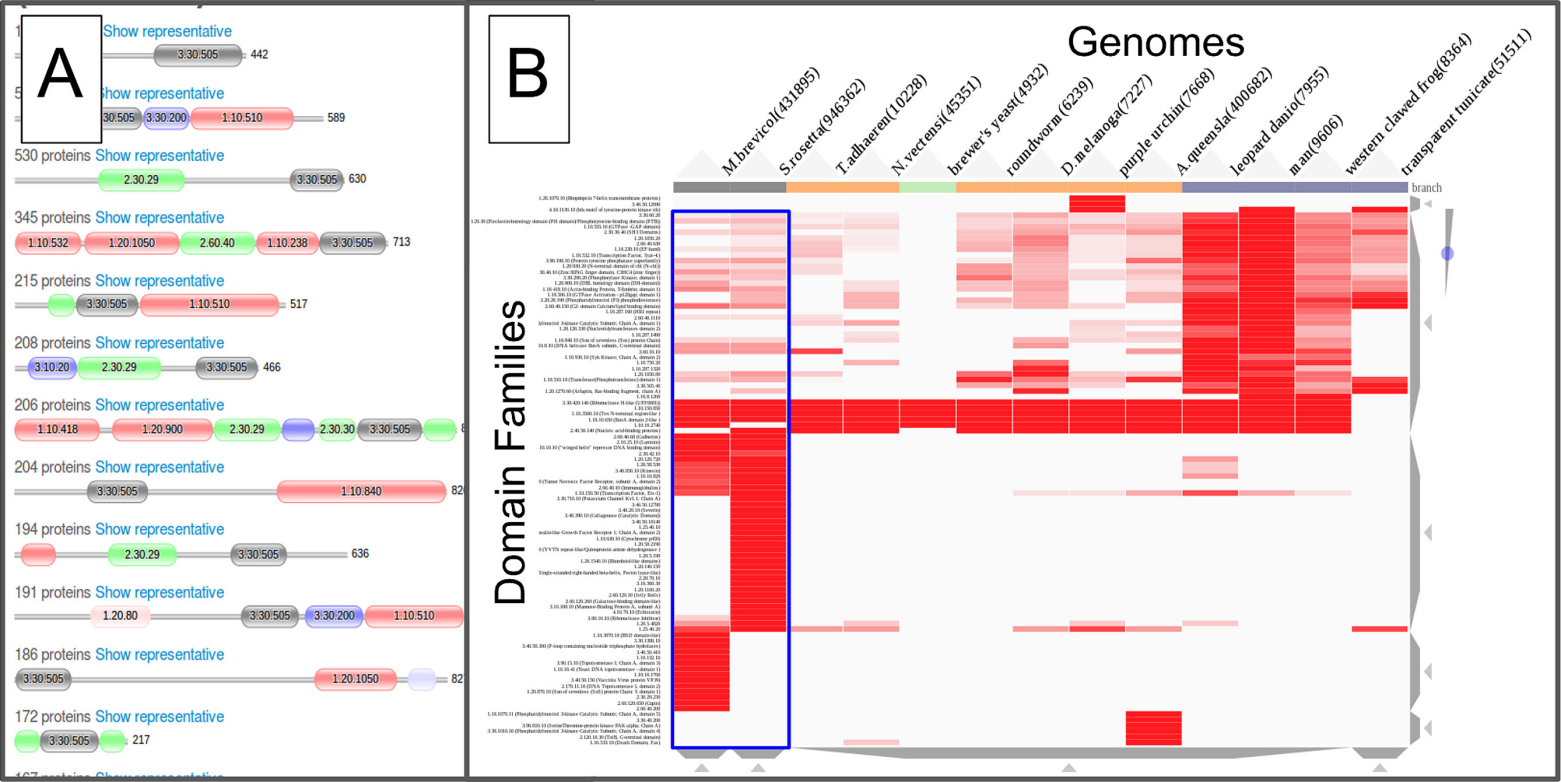
(**A**) The most frequent Domain Architectures that the SH2 domain family is found in amongst the Ensembl Genomes. (**B**) Gene3D domain family co-occurrence page, showing domain families that co-occur with the SH2 domain family in selected Eukaryotic and Metazoan genomes. The first two columns (surrounded by a blue box) correspond to two Choanoflagellate genomes. Darker red entries indicate a greater number of proteins in which the SH2 domain family co-occurs with a specific genome/domain family pair. The number of domain families displayed has been limited to the most populous 100 domain families for the selected genomes. Mousing over a point in the matrix gives popups with further detailed information.

The Domain Family pages show the representative domain architectures, ordered by the number of proteins in which they are predicted (Figure 3). The ordering can now be altered by the user, by specifying different taxonomic groupings (i.e. Plants, Protists, Fungi, Metazoa, Prokaryotes and representative Viral proteomes). As an example, we can look at the domain architectures for the SH2 domain family, specifically ordered by protein counts derived from Ensembl genomes (Figure 3A).

The Domain Architecture page also includes a table showing co-occurring domain families, grouped across different genomes. A table detailing counts for the query domain family with other domain families in specific taxonomic lineages can be found by scrolling down to the ‘Domain Partners’ section of the page. To look at how these co-occurring domain families compare across specific genomes, we have added another visualisation tool to the Gene3D website. This visualization tool displays the co-occurring domain families, for a given query domain family in a heatmap/matrix style, using the clustergrammer JavaScript software (https://github.com/MaayanLab/clustergrammer). These co-occurrence matrices, can get quite large, but the website offers various filters and the clustergrammer library allows the user to zoom in for details. Also, mousing-over individual rows and cells produces a popup with more detailed information. As an example, in the Gene3D website, we can search for domains that co-occur with the SH2 domain family (superfamily: 3.30.505.10) in a set of specified Eukaryotic and Metazoan genomes. The resulting page, shown in Figure 3B, has different genomes along the top (columns) and domain families on the left (rows). A cell (i.e. a specific row/column pair) being colored in red indicates that the domain family corresponding to that row is found in a protein that also has an SH2 domain family, in the genome corresponding to that column. In our example, we can see large changes in the domain families that the SH2 domain family co-occurs with (i.e. in the same proteins) in different genomes, see Figure 3B. In the Metazoan genomes, this represents the domain family being utilised in novel signalling functions, in multiple new contexts. Interestingly we can see that the SH2 domain families co-occurring partners are already very diverse in Choanoflagellate genomes (i.e. the first two columns highlighted by a blue box), the closest living relatives to Metazoans (Animals).

### Data downloads

We provide the Gene3D data and the domain assignment pipeline at the FTP site (ftp://orengoftp.biochem.ucl.ac.uk/gene3d/CURRENT_RELEASE/). We provide a number of genome annotations (e.g. UniProt Reference Proteomes) and can make bespoke datasets (e.g. a newly sequenced genome not in Gene3D) for download upon request. The package for scanning HMMs is provided along with instructions. Some example searches can be found by following the examples link (http://gene3d.biochem.ucl.ac.uk/examples).

## CONCLUSION

Improvements in techniques such as Cryo-EM (15), will lead to rapid expansions in the numbers and variety of atomic resolution 3D structures over the coming years. Whilst the structural and sequence datasets continue to grow, Gene3D keeps up with these expansions. The new Gene3D package is more portable and less memory intensive than the previous release and so should be more feasibly applicable by users for annotating their own datasets.

## Supplementary Material

Supplementary Data
