# Gene3D: Extensive prediction of globular domains in proteins

**DOI:** 10.1093/nar/gkx1187

**Published:** 2017-11-29

**Authors:** Tony E Lewis, Ian Sillitoe, Natalie Dawson, Su Datt Lam, Tristan Clarke, David Lee, Christine Orengo, Jonathan Lees

**Affiliations:** Institute of Structural and Molecular Biology, Division of Biosciences, University College London, Gower Street, London WC1E 6BT, UK; School of Biosciences and Biotechnology, Faculty of Science and Technology, University Kebangsaan Malaysia, 43600 Bangi, Selangor, Malaysia; Bristol Life Sciences Building, University of Bristol, Bristol Life Sciences Building, Bristol, BS8 1TQ, UK; Oxford Brookes University, Faculty of Health and Life Sciences, Oxford, Oxfordshire, UK

*Nucleic Acids Research* (2017), https://doi.org/10.1093/nar/gkx1069

The Authors would like to apologise for some small content errors in their text, including a mention on page 2 that the number of S35 domain sequences had been expanded from ‘2155 to over 32 000’. This should be ‘21 155 to over 32 000’. A spelling error in the name of Tony E. Lewis was also adapted. The paper has now been corrected online.

