# Supplementary figures and images for: Gene3D: Extensive prediction of globular domains in proteins
Source: Nucleic Acids Res. 2017 Nov 3;46(Database issue):D435–9. doi: 10.1093/nar/gkx1069 (PMC5753370; doi:10.1093/nar/gkx1069)

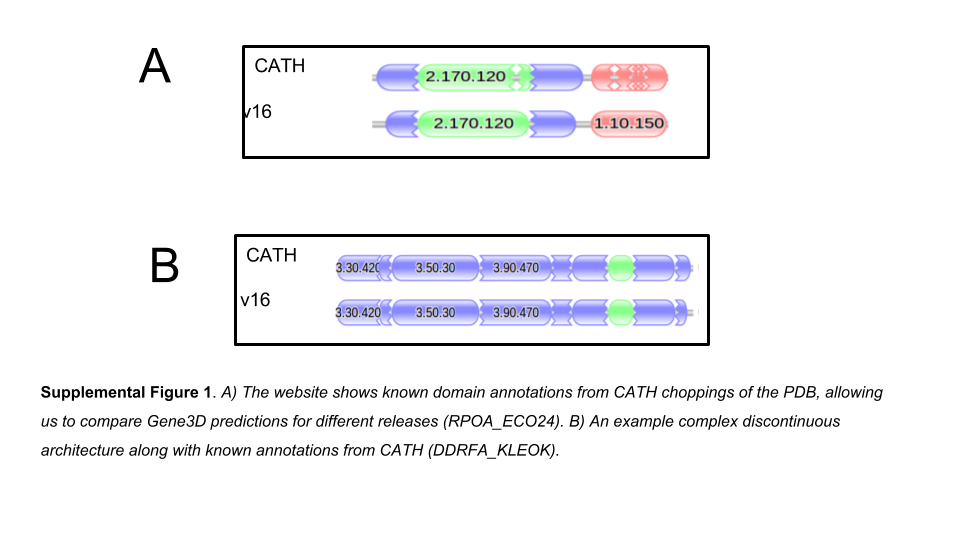

Supplement: Supplementary Data [file gkx1069_supp.png]
